# Supplementary material for: UV-B Physiological Changes Under Conditions of Distress and Eustress in Sweet Basil
Source: Plants (Basel). 2019 Oct 4;8(10):396. doi: 10.3390/plants8100396 (PMC6843199; doi:10.3390/plants8100396)

Supplemetary Information

Figure S1A Spectrum of white light emission from fluorescence tubes (L 36 W/76, Osram, Munich, Germany) provided by the Osram company.

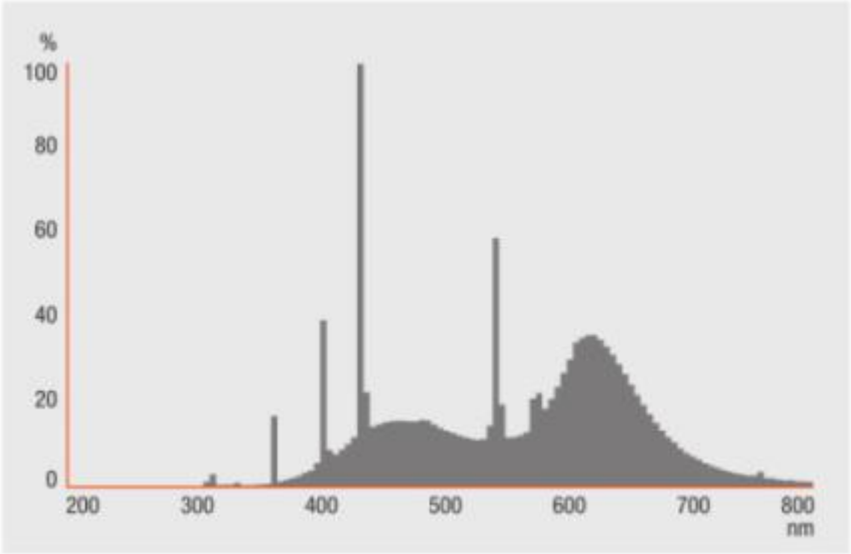

Figure S1B Spectrum of UV-B light emission from UV-B lamps (UVB GL20 SE, Sankyo Denki, Tokyo, Japan) provided by the Sankyo Denki company.

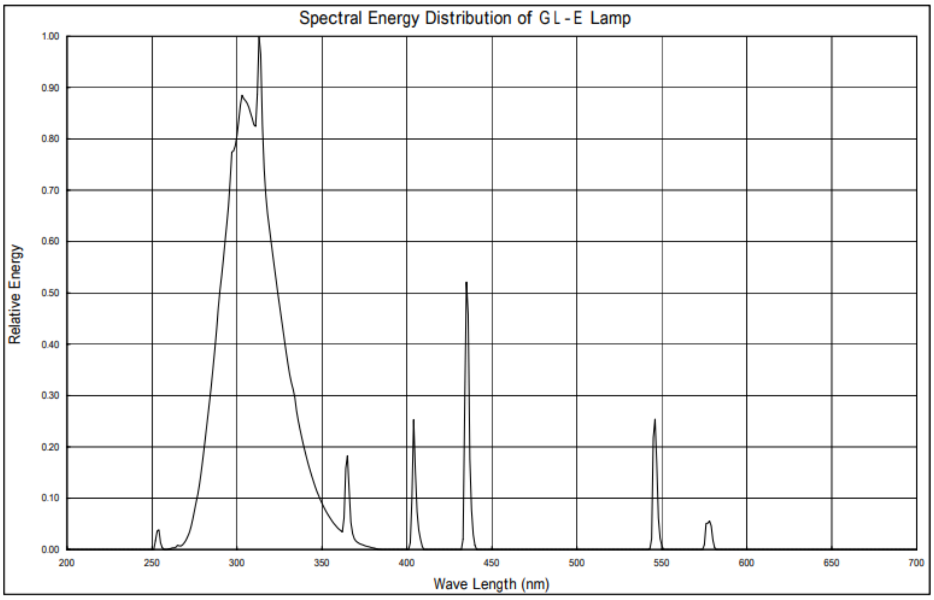

Supplement: Supplementary file 1 [file plants-08-00396-s001.pdf]
